# Soluble Receptor for Glycation End-products Concentration Increases Following the Treatment of Severe Diabetic Ketoacidosis

**DOI:** 10.4274/jcrpe.galenos.2019.2019.0076

**Published:** 2020-06-03

**Authors:** William H. Hoffman, Takaki Ishikawa, James Blum, Naoto Tani, Tomoya Ikeda, Carol M. Artlett

**Affiliations:** 1 Augusta University, Medical College of Georgia, Department of Pediatrics, Georgia, USA; 2 Osaka City University Faculty of Medicine, Department of Legal Medicine, Abeno Osaka, Japan; 3 University of North Carolina-Wilmington, Department of Mathematics and Statistics, North Carolina, USA; 4 Drexel University College of Medicine, Department of Microbiology and Immunology, Pennsylvania, USA

**Keywords:** Diabetic ketoacidosis, D-lactate, myocarditis, soluble receptor for advanced glycation end-products

## Abstract

**Objective:**

To determine the time relationships of soluble receptor for glycation end-products (sRAGE), [a decoy of the advanced glycation end-products (AGE)-RAGE axis] and D-lactate, (a metabolite of methylglyoxal) in the inflammatory response to diabetic ketoacidosis (DKA).

**Methods:**

Sixteen children and adolescents with type 1 diabetes (T1D) had blood samples obtained, 6-12 hours into treatment, at three weeks and three months post start of treatment. sRAGE and D-lactate concentrations at three months were considered baseline. Expression of RAGE was investigated in the myocardium of a newly diagnosed and untreated young person with fatal T1D/DKA.

**Results:**

sRAGE 6-12 hours after the start of treatment was 39% lower than the values at two weeks (p=0.0036) and at three months (p=0.0023) post treatment. D-lactate was higher during treatment than at three weeks (p=0.04) and at three months (p=0.035).

**Conclusion:**

sRAGE concentration was decreased during treatment, compared to concentrations at two weeks and three months after treatment. The increased D-lactate during treatment was in keeping with the known increase in dicarbonyls at this time. The finding of RAGE expression in a young myocardium prior to DKA treatment suggested cardiovascular inflammation pre-treatment and at a young age.

**What is already known on this topic?**

The advanced glycation end-products/receptor for glycation end-products (AGE-RAGE) axis is a significant factor in the pathogenesis of type 1 diabetes complications. It has been proposed that soluble RAGE may act in a protective role during diabetic ketoacidosis (DKA) episodes.

**What this study adds?**

This is the first study of its kind. A longitudinal study of DKA measuring the marker for AGE-RAGE, soluble RAGE (sRAGE) and examining the systemic pattern of this inflammatory pathway during DKA treatment. This inflammation was expressed very early in the heart tissue of a young person who died of DKA without treatment. This study again stresses the serious implications of even one episode of DKA.

## Introduction

Suboptimal metabolic control caused by the insulin deficiency of type 1 diabetes (T1D) involves varying degrees of metabolic and immunologic dysregulation, resulting in a milieu that mediates oxidative stress (1,2) and inflammation (3). With significant insulin deficits and poor control, this dysregulation leads to the medical crisis of diabetic ketoacidosis (DKA) and the increased potential of comorbidities. Prior to DKA there is a gradual/dysfunctional increase in an array of inflammatory cytokines, chemokines (4,5,6) and complement (7), followed by a systemic inflammatory response (SIR) shortly after the initiation of DKA treatment (4,8,9). The metabolic stress of hyperglycemia, hyperketonemia and increased reactive oxygen species also initiates the non-enzymatic glycosylation of glucose with free amino acids to form the toxic α-dicarbonyls (10,11). These precursors/intermediates lead to the formation of advanced glycation end products (AGEs), ligands for the receptor of AGE (RAGE) and for soluble RAGE (sRAGE) (12). RAGE is ubiquitous, and has a major role in the pathogenesis of diabetic cardiovascular comorbidities, even in newly diagnosed patients with diabetes (13,14). sRAGE is a proteolytic, cleaved, secretory isoform, a natural competitor of RAGE and is a protective “decoy” that abrogates the insults that otherwise occur as a result of AGE ligands transferring to, binding to and activating RAGE (13).

Despite impressive advances in understanding the pathogenesis of the AGE-RAGE axis in acute and chronic medical conditions, uncertainties remain in the pathogenesis of T1D comorbidities and in DKA (15), a relative frequent medical crisis in children and adolescents (16). The recent article by Rawshani et al (17) gives reason to reconsider the seriousness of poorly controlled T1D in terms of longevity in children, even though DKA is not referred to. The importance of DKA can be deduced because of its common occurrence when the age of onset is before 10 years, and with the resulting loss of approximately 15 life-years for both women and men. This unfortunate statistic does not fully consider quality of life, including achievement, a factor that is much more difficult to quantify.

This data prompted us to examine the systemic inflammatory marker sRAGE during and after DKA treatment when an increase of toxic and inflammatory factors, such as the dicarbonyls and inflammatory cytokines, are expressed at the same approximate times (4,5,8,10,11). D-lactate was used as the metabolic marker of flux or catabolism of methylglyoxal (MG) (18), the precursor for the AGE ligands hydroimidazolone-1 (MG-H1), the most abundant human AGE; and N(epsilon)-(carboxymethyl) lysine (19). The myocardial expression of RAGE was also investigated in an undiagnosed and untreated, fatal case of T1D/DKA (20) to give insight into: 1) the role of treatment in RAGE expression; and 2) the likely developmental sequence of chronic cardiovascular complications of RAGE that result from severe DKA.

## Methods

### Study Design and Patients

A prospective longitudinal study design was utilized to study a cohort of children and adolescents with T1D/DKA. The study received Expedited Approval by the institutional review board at East Carolina University (ECU) Brody School of Medicine, since blood samples were only obtained at the time of routine blood sampling for treatment of DKA and follow up visits. The study was conducted in accordance with the Declaration of Helsinki. A total of sixteen children and adolescents between the ages of 9.5 and 17 years, presenting with DKA (total CO_2_=/<12 mmol/L) were invited to enroll in the study. Informed consent was signed by the legal guardian and assent was obtained from the patients of nine years and over, when not prohibited by severity of illness. In such cases, patient assent was obtained when clinical improvement permitted. Patients referred from outlying hospitals were stabilized prior to being transported to ECU after consultation with the accepting attending physician in the Pediatric Intensive Care Unit. Treatment was according to previously published guidelines (21) with each patient serving as their own control and the three-month follow-up time point (T3) served as the baseline. Transfer of children and adolescents for treatment of DKA was routine in this part of North Carolina at the time of the study.

### Study Evaluation and Analysis

Pretreatment values were obtained for blood pressure (BP), heart rate, complete blood count (CBC), blood glucose (BG), electrolytes, urea nitrogen (BUN), and creatinine at the referring hospitals. The start of treatment was defined as the initiation of continuous intravenous insulin. In addition to the pretreatment BP, BPs were recorded hourly with an automated oscillometric device and appropriately sized BP cuff. BPs were also obtained hourly after initiation of treatment) (T1); at discharge; two weeks post discharge (T2); and at baseline which was three months post discharge (T3). BG measurements were obtained hourly, electrolytes, and BUN were measured every two to four hours. A CBC and differential was repeated at 24 hours. None of the patients were known to have hypertension, diabetic retinopathy, nephropathy or coronary artery disease. Exclusion criteria included a history or physical findings suggestive of an acute or chronic infection, emotional or physical disability or autoimmune conditions other than chronic lymphocytic thyroiditis.

Serum samples were analyzed undiluted using an enzyme-linked immunoabsorbent assay according to the manufacturer’s instructions (Human RAGE ELISA, R&D Systems, Minneapolis, MN., USA). The inter-assay coefficient of variation was 7.6%, while the intra-assay coefficient of variation was 3.5%. The analysis included the pool of both circulating esRAGE and sRAGE.

Serum D-lactate was measured by kinetic spectrophometric assay, using the D-lactate Colormetric Assay kit MAK058 (Sigma, St. Louis, MO., USA). D-lactate was employed as it is the end-product of MG catabolism by glyoxalase 1 and 2. In this method, D-lactate is specifically oxidized by bacterial D-lactate dehydrogenase (LDH). To increase the sensitivity of the assay we incubated samples at 37 °C and the reaction was followed kinetically to achieve maximal sensitivity and linearity. To eliminate interference by the reaction of serum L-LDH with L-lactate, serum was ultrafiltered. The <10 kDA fraction was separated by ultrafiltration through 0.5 mL Amicon Ultra Centrifugal filters spun at 14,000 g for 30 minutes in a refrigerated centrifuge at 4 °C. The ultrafiltrate was used to measure D-lactate. The limit of detection was 1 µM and the reaction was linear up to 15 µM. The intra-assay CV at 2 µM and 10 µM was 5% and 3% respectively. To further ensure specificity, the reaction was performed with and without 1 mmol/l L-lactate (upper limit of reference range in serum) and identified <5% interference (p<0.05), in agreement with data in the literature.

Immunohistochemistry (IHC) for myocardial RAGE was studied in the left ventricles of the undiagnosed and untreated case of DKA and the control. Sections were deparaffinized in two changes of xylene and two changes of absolute ethanol for 10 minutes each. Antigen retrieval was performed in 10 mM buffer, pH 6.0 in a microwave for 30 minutes then rinsed in phosphate buffered saline (PBS). Sections were blocked in 5% donkey serum for 20 minutes, then PBS for two minutes. Rabbit anti-RAGE, diluted 1:1000 (GeneTex, Irvine, CA., USA), was applied for 40 minutes at room temperature in a humid chamber, then unbound antibody removed with three changes of PBS for two minutes each. The secondary antibody, donkey anti-rabbit-Cy3, diluted 1:1,500 was then applied for 40 minutes at room temperature in a humid chamber. Unbound antibody was removed with three further changes of PBS for two minutes each. The sections were then counterstained with Dapl and viewed with an epi fluorescent microscope. All images were documented using the same magnification (x200).

### Statistical Analysis

Normality of the data was determined using the Shapiro-Wilk test. For the variables that did not show a normal distribution they are described with median and interquartile range (IQR). Tests for differences between [A (T1), B (T2) and C (T3) (baseline/3 months post-discharge)] in least-square means of RAGE and D-lactate scores were performed with a repeated measures ANOVA model, with the Tukey adjustment for multiple tests applied to the p values. A correlation analysis of Spearman was used to determine relationships between variables. Results were considered significant with p<0.05. Statistical analyses were performed using the SPSS software statistical package for Mac, version 19.0 (SPSS, Chicago, IL, USA).

## Results

The cohort was representative of the middle Eastern coast of North Carolina. The median age of the 16 patients was 13.6 (9.7-16.9) years. The mean duration of T1D for the 10 previously diagnosed patients was 5.7 (1-12) years. Six patients were newly diagnosed with T1D at the time of admission (duration one day). There were seven males and nine females; six Caucasians (C) and 10 African Americans (AA). Patients were within 2 standard deviation (SD) of their height for age and had weights within 1.5 SD of their ideal weight for height (data not shown). All patients had uneventful correction of DKA (neurocognitive testing was not performed). All patients had at least one positive islet cell autoantibody test (IAA, IA-2 and GAD65; data not shown).

### Laboratory Findings in Patients

At T1 the following biochemistry results (median and IQR) were found: BG 26.6 (14.4-46.9) mmol/L which at discharge had fallen to 10 (5.3-12.2) mmol/L; sodium 135.8 (130-144) mmol/L; potassium 5.2 (3.9-6.7) mmol/L; chloride 100.3 (90-111) mmol/L; total CO_2_ 10.5 (9-11) mmol/L; and BUN 6.4 (4.3-15) mmol/L. None of these admission parameters, other than the increased BG concentration, had significant associations with the studied metabolic inflammatory markers (see below).

Median (IQR) sRAGE concentration (pg/mL) was significantly lower at T1 at 332.18 (257.85-506.85) compared with T3 546.20 (390.42-739.19) (p=0.0023), representing a 39% difference (Table 1). There was a strong negative correlation between the decreased sRAGE concentration (T1) and increased BG concentration [r=-0.59; p=<0.0001). sRAGE concentration at T3 was higher in:

1) Females vs males 237.3 (176.4-446.2) vs 156.5 (76.4-191.8) pg/mL, p=0.04;

2) C vs AA at 867.9 (585.0-1,243.1) vs 459.7 (356.1-546.2) pg/mL, p=0.003; and

3) For the patients with newly diagnosed T1D/DKA vs previously diagnosed patients with DKA at 721.6 (585-768.1) vs 549.7 (356.1-571.8) pg/mL, p=0.04.

D-lactate concentrations significantly (p=0.035) decreased from T1 (14.1 µmol/L) to T3 (3.7 µmol/L). There was also a negative correlation which approached significance between the increased D-lactate concentration and decreased sRAGE concentration at T1 (r=-0.32; p=0.05). The D-lactate at T3 of newly diagnosed patients with T1D/DKA was significantly lower compared to patients who had an existing diagnosis of T1D but presented with DKA [2.3 (1.2-2.8) vs 8.6 (3.2-15.9) µmol/L; p=0.04] (Table 1).

### Tissue Expression of RAGE in Myocardium

**Figure 1:**
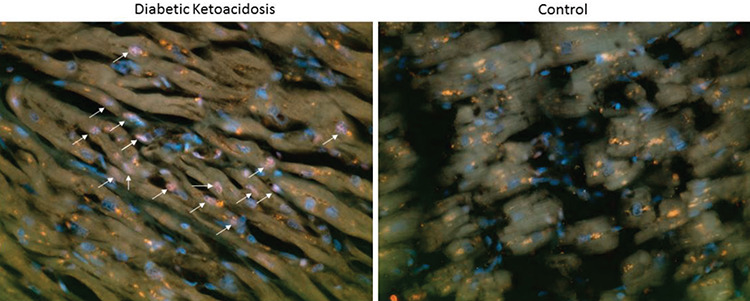
RAGE was prominently expressed in the diabetic ketoacidosis myocardium versus the gender and age matched control myocardium. RAGE expression in the myocardium (left ventricle) of a young, Japanese woman, aged approximately 22 years, who died of undiagnosed (new onset) T1D/DKA prior to treatment. The arrows show positively stained RAGE cells. No stain was present in the age and gender matched control. Magnification x200

The young woman with undiagnosed T1D/DKA was found dead in her apartment, approximately 24 hours after her death. She was approximately 22 years old with a height of 156 cms weight of 37.3 kg and a BMI of 15.3 (20). IHC of myocardial RAGE expression is shown in Figure 1. Compared to control tissue, there was marked albeit subjective increase in the staining for RAGE in the myocardial samples of the young woman with undiagnosed T1D/DKA.

## Discussion

To the best of our knowledge this is the first longitudinal report of sRAGE concentrations during and after the correction of severe DKA. We report a 39% lower concentration of sRAGE during DKA treatment (T1; 6-12 hours into treatment) compared with three months after the episode (p=0.0023). There was also a significant increase in sRAGE at T1 compared with T2, two weeks post treatment, (p=0.0036). The trend to increase in sRAGE concentrations continued to the final study point at three months, however there was no significant difference between concentrations of sRAGE measured at T2 and T3 (p=NS). We hypothesize that the decrease in the sRAGE (decoy) concentrations at T1 occurred early in, or possibly prior to, DKA treatment as the result of the significantly increased concentrations of dicarbonyls (10,11), with accompanying AGE ligand formation followed by sRAGE sequestration. This sequence minimizes or prevents ligand binding, and activation of RAGE. Depending on the extent of the initial ligand binding both mediators of capillary perturbation -MG and MGH1- are candidates to be involved in the pathogenesis of pretreatment subclinical brain edema (22) and interstitial pulmonary edema (23) that occurs in severe DKA. In this regard an additional ligand of sRAGE, malondialdehyde, which is a highly reactive and a damaging β dicarbonyl, is also elevated during DKA (24), resulting in lipid peroxidation, breakdown of phospholipids, and increased vascular endothelial permeability (25,26). These pretreatment subclinical capillary perturbations are relatively common (27) and can progress during DKA treatment, but rarely to the extent of causing signs/symptoms (22,28). A decrease of early vascular perturbators at the time of treatment of severe DKA is in keeping with the hypotheses of Grossin et al (29) and Salonen et al (30), which is that sRAGE has a protective effect. With the decrease of sRAGE and its ligand sequestrations, the residual unsequestered ligand can then activate RAGE.

D-lactate, the steroisomer of L-lactate, the other metabolic marker studied, was formerly viewed to be a metabolic by-product, but is now recognized as an active metabolite in signaling of pro-inflammatory circuits. In particular D-lactate controls T-cell migration (31) and also contributes to the anion gap in the metabolic acidosis of DKA. This marker of MG catabolism was increased at 6-12 hours (T1), and decreased two weeks following treatment (T2) (p=0.04), with a further decrease at three months (T3) (p=0.035). This systemic pattern of D-lactate is in keeping with an early decrease of sRAGE and later, subclinical perturbation of the myocardium (5). A serious effect of D-lactate is its limited ability to be an effective respiratory substrate in the rat heart and brain due to its altering of mitochondrial energy production (32). This raises the question: Does a lower D-lactate concentration act synergistically with other DKA perturbations resulting in subclinical cardiac insults?

The longitudinal study of pre-diabetic children by Salonen et al (30) reported a decrease of sRAGE prior to the seroconversion to positive pancreatic autoantibodies. Both this and the present DKA study document low sRAGE in relation to DKA insult. The decrease of sRAGE in the study of Salonen et al (30) was reported to occur approximately 30 days before the onset of DKA with no further sRAGE decrease. In contrast, our study identifies a period of sRAGE increase following DKA treatment. We believe these transitions could be influenced by a gradual change in the pH of the milieu. However, low sRAGE concentrations are reported to occur in various conditions during the acute clinical phase in addition to changes reported in DKA. These conditions include: 1) atrial fibrillation (33); 2) low sRAGE and high cardiac troponin in non-ST segment elevation myocardial infarction (34); and 3) the autoimmune condition of multiple sclerosis (35).

Our study was not intended to identify a cause and effect relationship between the two inflammatory pathways of the AGE-RAGE axis and the SIR of inflammatory cytokines, but rather, was intended to compare their systemic phenotypes during the treatment of DKA, a time of known myocardial perturbation (5,36) and post treatment. This difference between the two inflammatory pathways was evident during the 6-12 hour period of treatment (T1) with sRAGE being lower in comparison to the rapid increase in the majority of cytokines, possibly initiated by insulin treatment (4,5,8,9). While interactions between components of the two pathways are likely (37,38,39), we were unable to confirm interactions in this study. Of interest, we also noted a difference in sRAGE concentrations at three months post-DKA therapy by ethnicity and gender with C having significantly higher (p=0.003) sRAGE concentrations compared with AA and this concentration was also significantly higher (p=0.04) in females than males. However, the small sample size means that although these findings are interesting, they remain to be confirmed in larger cohorts.

Despite the dichotomy in systemic inflammatory patterns at approximately the same time during treatment, the transient systemic decrease of sRAGE and the rapid transient increase of inflammatory cytokines of the SIR (4) likely occurs shortly after (IV) insulin is initiated. In regard to this dichotomy of two systemic inflammatory patterns, including RAGE, it is of note that both inflammatory pathways have significant histologically proven expression in teenage brains following fatal DKA/BE (40,41,42). This pattern of brain expression is similar to the positive association between these two inflammatory systemic pathways reported in adults with T2D adults and may be unrelated to DKA (43).

The significant, myocardial IHC expression of the multiligand receptor RAGE in the young woman found “dead-in-bed” with new onset T1D/DKA (20) suggests early RAGE-mediated cellular activation and a positive feedback initiated by sRAGE-ligand and RAGE interaction. This expression is in keeping with a report of two young fatal T1D/DKA cases (deletion of phrase) both of whom had significant myocardial expression of the inflammatory markers MCP-1 and IL-1 beta (44). These autopsy IHC studies, along with the synthesis of cardiac autoantibodies in uncomplicated severe DKA (36), support the hypothesis that subclinical myocarditis can be initiated by the inflammatory insults of the AGE-RAGE axis and by inflammatory cytokines in T1D/DKA, with eventual progression to diabetic cardiomyopathy in some T1D patients. Yang et al (45) reported that blocking of RAGE attenuates autoimmune myocarditis which is additional supportive evidence for this hypothesis.

## Conclusion

While the limitation of this study is the size of the patient cohort, this study adds to the evidence that the AGE-RAGE axis is a contributor to the acute inflammatory insult during the medical crisis and treatment of DKA and acts as constant source of subclinical inflammation leading to chronic diabetic vascular complications, including those of the heart. Inflammation during DKA treatment involved a significant transient decrease of sRAGE, possibly prior to treatment, and a significant transient increase of D-lactate, both metabolic markers of AGE-RAGE activity. It is suggested that, following the dissipation of systemic sRAGE, RAGE expression would increase and D-lactate decrease. This pattern would supports the hypothesis of sRAGE being a protective “decoy” prior to cell perturbation, through sequestration of its ligand, RAGE (10,11,31). The finding of lower concentrations of sRAGE both for AAs compared to Cs, and for males compared to females at T3, and with the opposite relationship reported for inflammatory cytokines during severe uncomplicated DKA treatment (5) warrant further investigation with larger sample sizes. The significant myocardial RAGE expression of the young woman who died of undiagnosed and untreated DKA (20) adds to the previously reported increase in myocardial inflammatory cytokines in young patients who died during the treatment of severe DKA (44). Myocardial expression of RAGE suggests a further pathogenetic mechanism for the AGE-RAGE axis, unrelated to DKA treatment (20). Whether RAGE only becomes activated in the myocardium during the life-threatening crisis of severe DKA and whether early myocardial RAGE expression occurs in less severe forms of metabolic/immunologic DKA insults, in addition to the relationship with cardiac function, all require careful follow-up and further study.

## Figures and Tables

**Table 1:**
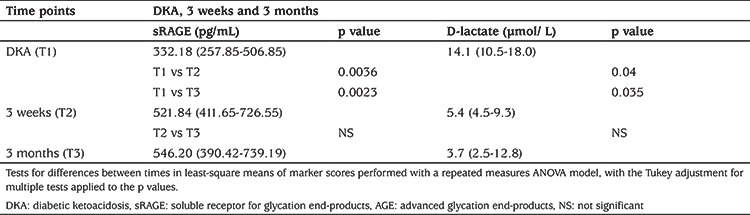
Comparison of median sRAGE and D-lactate concentrations between the different time points examined in the study

## References

[1] Yao D, Brownlee M (2010). Hyperglycemia-induced reactive oxygen species increase expression of the receptor for advanced glycation end products (RAGE) and RAGE ligands. Diabetes.

[2] Lee DM, Hoffman WH, Carl GF, Khichi M, Cornwell PE (2002). Lipid peroxidation and anti-oxidant vitamins prior to, during and after correction of diabetic ketoacidosis. J Diabetes Complications.

[3] Heier M, Margeirsdottir HD, Brunborg C, Hanssen KF, Dahl-Jorgensen K, Seljeflot I (2015). Inflammation in childhood type 1 diabetes; influence of glycemic control. Atherosclerosis.

[4] Hoffman WH, Burek CL, Waller JL, Fisher LE, Khichi M, Mellick LB (2003). Cytokine response to diabetic ketoacidosis and its treatment. Clin Immunol.

[5] Hoffman WH, Passmore GG, Hannon DW, Talor MV, Fox P, Brailer C, Haislip D, Keel C, Harris G, Rose NR, Fiordalisi I, Cihakova D (2013). Increased systemic Th17 cytokines are associated with diastolic dysfunction in children and adolescents with diabetic ketoacidosis. PLoS One.

[6] Omatsu T, Cepinskas G, Clarson C, Patterson EK, Alharfi IM, Summers K, Couraud PO, Romero IA, Weksler B, Fraser DD;, Canadian Critical Care Translational Biology Group (2014). CXCL1/CXCL8 (GROα/IL-8) in human diabetic ketoacidosis plasma facilitates leukocyte recruitment to cerebrovascular endothelium in vitro. Am J Physiol Endocrinol Metab.

[7] Jerath RS, Burek CL, Hoffman WH, Passmore GG (2005). Complement activation in diabetic ketoacidosis and its treatment. Clin Immunol.

[8] Karavanaki K, Karanika E, Georga S, Bartzeliotou A, Tsouvalas M, Konstantopoulos I, Fotinou A, Papassotiriou I, Karaylanni C (2011). Cytokine response to diabetic ketoacidosis (DKA) in children with type 1 diabetes (T1DM). Endocr J.

[9] Close TE, Cepinskas G, Omatsu T, Rose KL, Summers K, Patterson EK, Fraser DD (2013). Diabetic ketoacidosis elicits systemic inflammation associated with cerebrovascular endothelial dysfunction. Microcirculation.

[10] Hoffman WH, Kappler F, Passmore GG, Mehta R (2003). Diabetic ketoacidosis and its treatment increase plasma 3-deoxyglucosone. Clin Biochem.

[11] Turk Z, Nemet I, Varga-Defteardarovic L, Car N (2006). Elevated level of methylglyoxal during diabetic ketoacidosis and its recovery phase. Diabetes Metab.

[12] Degenhardt TP, Thorpe SR, Baynes JW (1998). Chemical modification of proteins by methylglyoxal. Cell Mol Biol.

[13] Hori O, Yan SD, Ogawa S, Kuwabara K, Matsumoto M, Stern D, Schmidt AM (1996). The receptor for advanced glycation end-products has a central role in mediating the effects of advanced glycation end-products on the development of vascular disease in diabetes mellitus. Nephrol Dial Transplant.

[14] Villegas-Rodriquez ME, Uribarri J, Solorio-Meza SE, Fajardo-Araujo ME, Cai W, Torres-Graciano S, Rangel-Salazar R, Wrobel K, Garay-Sevilla ME (2016). The AGE-RAGE axis and its relationship to markers of cardiovascular disease newly diagnosed diabetic patients. PLoS One.

[15] Koyama H, Yamamoto H, Nishizawa Y (2007). RAGE and soluble RAGE: potential therapeutic targets for cardiovascular diseases. Mol Med.

[16] Maahs DM, Hermann JM, Holman N, Foster NC, Kapellen TM, Allgrove J, Schatz DA, Hofer SE, Campbell F, Steigleder-Schweiger C, Beck RW, Warner JT, Holl RW;, National Paediatric Diabetes Audit and the Royal College of Paediatrics and Child Health, the DPV Initiative, and the T1D Exchange Clinic Network (2015). Rates of diabetic ketoacidosis: International comparison with 49,859 pedatric patients with type 1 diabetes from England, Wales, the U.S., Austria, and Germany. Diabetes Care.

[17] Rawshani A, Sattar N, Franzen S, Rawshani A, Hattersley AT, Svensson AM, Eliasson B, Gudbjornsdottir S (2018). Excess mortality and cardiovascular disease in young adults with type 1 diabetes in relation to age at onset: a nationwide, register based cohort study. Lancet.

[18] Misra K, Banerjee AB, Ray S, Ray M (1995). Glyoxalase III from Escherichia coli: a single novel enzyme for the conversion of methylglyoxal into D-lactate without reduced glutathione. Biochem J.

[19] Heier M, Margeirsdottir HD, Torjesen PA, Seljeflot I, Stensaeth KH, Gaarder M, Brunborg C, Hanssen KF, Dahl-Jorgensen K (2015). The advanced glycation end product methylglyoxal-derived hydroimidazolone-1 and early signs of atherosclerosis in childhood diabetes. Diab Vasc Dis Res.

[20] Tani N, Michiue T, Chen JH, Oritani S, Ishikawa T (2016). Usefulness of postmortem biochemistry in identification of ketosis: Diagnosis of ketoacidosis at the onset of autoimmune type 1 diabetes in an autopsy case with cold exposure and malnutrition. Legal Med (Tokyo).

[21] Fiordalisi I, Novotny WE, Holbert D, Finberg L, Harris GD;, Critical Care Management Group (2007). An 18 year prospective study of pediatric diabetic ketoacidosis an approach to minimizing the risk of brain herniation during treatment. Pediatr Diabetes.

[22] Hoffman WH, Steinhart CM, el Gammal T, Steele S, Cuadrado AR, Morse PK (1988). Cranial CT in children and adolescents with diabetic ketoacidosis. Am J Neuroradiol.

[23] Hoffman WH, Locksmith JP, Burton EM, Hobbs E, Passmore GG, Pearson-Shaver AL, Deane DA, Beaudreau M, Bassali RW (1998). Interstitial pulmonary edema in children and adolescents with diabetic ketoacidosis. J Diabetes Complications.

[24] Vantyghem MC, Balduyck M, Zerimech F, Martin A, Douillard C, Bans S, Degand PM, Lefebvre J (2000). Oxidative markers in diabetic ketoacidosis. J Endocrinol Invest.

[25] Samsonov MV, Khapchaev AY, Vorotnikov AV, Vlasik TN, Yanushevskaya EV, Sidorova MV, Efremov EE, Lankin VZ, Shirinsky VP (2017). Impact of atherosclerosis- and diabetes- related dicarbonyls on vascular endothelial permeability: A comparative assessment. Oxid Med Cell Longev.

[26] Reis JS, Veloso CA, Volpe CM, Fernandes JS, Borges EA, Isoni CA, Dos Anjos PM, Nogueira-Machado JA (2012). Soluble RAGE and malondialdehyde in type 1 diabetes patients without chronic complications during the course of the disease. Diab Vasc Dis Res.

[27] Glaser NS, Wootton-Gorges SL, Buonocore MH, Marcin JP, Rewers A, Strain J, DiCarlo J, Neely EK, Barnes P, Kuppermann N (2006). Frequency of sub-clinical cerebral edema in children with diabetic ketoacidosis. Pediatr Diabetes.

[28] Wolfsdorf J, Craig ME, Daneman D, Dunger D, Edge J, Lee WR, Rosenbloom A, Sperling MA, Hanas R;, International Society for Pediatric and Adolescent Diabetes (2007). Diabetic ketoacidosis. Pediatr Diabetes.

[29] Grossin N, Wautier MP, Meas T, Guillausseau PJ, Massin P, Wautier JL (2008). Severity of diabetic microvascular complications is associated with a low soluble RAGE level. Diabetes Metab.

[30] Salonen KM, Ryhanen SJ, Forbes JM, Borg DJ, Harkonen T, Ilonen J, Simell O, Veljola R, Groop PH, Knip M (2015). Decrease in circulating concentrations of soluble receptors for advanced glycation end products at the time of seroconversion to autoantibody positivity in children with prediabetes. Diabetes Care.

[31] Haas R, Smith J, Rocher-Ros V, Nadkarni S, Montero-Melendez T, D’Acquisto F, Bland EJ, Bombardieri M, Pitzalis C, Perretti M, Marelli-Berg FM, Mauro C (2015). Lactate regulates metabolic and proinflammatory circuits in control of T cell migration and effector functions. PLoS Biol.

[32] Ling B, Peng F, Alcorn J, Lohmann K, Bandy B, Zello GA (2012). D-lactate altered mitochondrial energy production in rat brain and heart but not liver. Nutr Metab (London).

[33] Al Rifai M, Schneider AL, Alonso A, Maruthur N, Parrinello CM, Astor BC, Hoogeveen RC, Soliman EZ, Chen LY, Ballantyne CM, Halushka MK, Selvin E (2015). sRAGE, inflammation and risk of atrial fibrillation: results from the Atherosclerosis Risk in Communities (ARIC) Study. J Diabetes Complications.

[34] McNair ED, Wells CR, Qureshi AM, Pearce C, Caspar-Bell G, Prasad K (2011). Inverse association between cardiac troponin-1 and soluble receptor for advanced glycation end products in patients with non-ST-segment elevation myocardial infarction. Int J Angiol Winter.

[35] Glasnovic A, Cvija H, Stojic M, Tudoric-Deno I, Ivcevic S, Romic D, Ticinovic N, Vuletic V, Lazibat I, Grcevic D (2014). Decreased level of sRAGE in the cerebrospinal fluid of multiple sclerosis patients at clinical onset. Neuroimmunomodulation.

[36] Hofffman WH, Sharma M, Cihakova D, Talor M, Rose NR, Mohanakumar T (2016). Cardiac autoantibody production to self-antigens in children and adolescents during and following the correction of severe diabetic ketoacidosis. Autoimmunity.

[37] McNair ED, Wells CR, Mabood Qureshi A, Basran R, Pearce C, Orvold J, Devilliers J, Prasad K (2010). Modulation of high sensitivity C-reactive protein by soluble receptor for advanced glycation end products. Mol Cell Biochem.

[38] Serban AI, Stanca L, Geicu OI, Dinischiotu A (2015). AGEs-induced IL-6 synthesis preceeds RAGE up-regulation in HEK 293 cells: an alternative inflammatory mechanism?. Int J Mol Sci.

[39] Son S, Hwang I, Han SH, Shin JS, Shin OS, Yu JW (2017). Advanced glycation end products impair NLRP3 inflammasome-mediated innate immune responses in macrophages. J Biol Chem.

[40] Hoffman WH, Cudrici CD, Zakranskaia E, Rus H (2006). Complement activation in diabetic ketoacidotic brains. Exp Mol Pathol.

[41] Hoffman WH, Casanova MF, Cudrici CD, Zakranskaia E, Venugopalan R, Nag S, Oglesbee MJ, Rus H (2007). Neuroinflammatory response of the choroid plexus epithelium in fatal diabetic ketoacidosis. Exp Mol Pathol.

[42] Hoffman WH, Artlett CM, Zhang W, Kreipke CW, Passmore GG, Rafols JA, Sima AA (2008). Receptor for advanced glycation end products and neuronal deficit in the fatal brain edema of diabetic ketoacidosis. Brain Res.

[43] Niu J, Gilliland MG, Jin Z, Kolattukudy PE, Hoffman WH (2014). MCP-1 and IL-1B expression in the myocardium of two young patients with Type 1 diabetes mellitus and fatal diabetic ketoacidosis. Exp Mol Pathol.

[44] Nakamura K, Yamagishi S, Adachi H, Kurita-Nakamura Y, Matsui T, Yoshida T, Imaizumi T (2007). Serum levels of sRAGE, the soluble form of receptor for advanced glycation end products, are associated with inflammatory markers in patients with type 2 diabetes. Mol Med.

[45] Yang WI, Lee D, Lee DL, Hong SY, Lee SH, Kang SM, Choi DH, Jang Y, Kim SH, Park S (2014). Blocking the receptor for advanced glycation end product activation attenuates autoimmune myocarditis. Circ J.

